# Rethinking the soil core microbiome

**DOI:** 10.1111/nph.71509

**Published:** 2026-08-17

**Authors:** Jaejin Lee, Bolívar Aponte Rolón, Phillip de Lorimier, Jean‐Michel Ané, Gian Maria Niccolò Benucci, Alyssa A. Carrell, Yen Ning Chai, Janith Chandrasoma, Nicole Geerdes, Valentina Infante, Brandon Kristy, Stanton Martin, Imani Pascoe, Rounak Patra, Lorien Radmer, Tomás Allen Rush, Ndenum Suzzy Shita, Andie Suratt, Patrick F. Suthers, Melissa A. Cregger, Adina Howe, Sarah Lebeis, Henrik Vibe Scheller

**Affiliations:** ^1^ Department of Agricultural and Biosystems Engineering Iowa State University Ames IA 50011 USA; ^2^ DOE Center for Advanced Bioenergy and Bioproducts Innovation University of Illinois Urbana‐Champaign Urbana IL 61801 USA; ^3^ The Great Lakes Bioenergy Research Center University of Wisconsin‐Madison Madison WI 53726 USA; ^4^ Department of Bacteriology University of Wisconsin‐Madison Madison WI 53706 USA; ^5^ Department of Plant and Agroecosystem Sciences University of Wisconsin‐Madison Madison WI 53706 USA; ^6^ Department of Plant, Soil and Microbial Sciences Michigan State University East Lansing MI 48824 USA; ^7^ Biosciences Division Oak Ridge National Laboratory Oak Ridge TN 37831 USA; ^8^ The Center for Bioenergy Innovation Oak Ridge TN 37831 USA; ^9^ Environmental Genomics and Systems Biology Division Lawrence Berkeley National Laboratory Berkeley CA 94720 USA; ^10^ Joint BioEnergy Institute Berkeley CA 94608 USA; ^11^ Carl R. Woese Institute for Genomic Biology University of Illinois Urbana‐Champaign Urbana IL 61801 USA; ^12^ Department of Integrative Biology Michigan State University East Lansing MI 48824 USA; ^13^ Department of Microbiology, Genetics, and Immunology Michigan State University East Lansing MI 48824 USA; ^14^ Department of Genetics University of Georgia Athens GA 30602 USA; ^15^ Department of Chemical Engineering The Pennsylvania State University University Park PA 16802 USA; ^16^ Plant Resilience Institute Michigan State University East Lansing MI 48824 USA

**Keywords:** bioenergy crops, core microbiome, explanatory subsets of taxa, functional convergence, rhizosphere microbiome, soil microbiome, taxonomic conservation

## Abstract

The concept of a core microbiome emerged from host‐associated research to describe microbial members or functions conserved across clearly defined spatial, temporal, and biological boundaries. In soil‐ and plant‐associated microbiome research, however, the term has increasingly shifted toward analytically defined subsets selected using study‐specific thresholds or criteria. Synthesizing recent literature and cross‐site analyses of bioenergy crop field soils, we show that the original biological meaning of the core microbiome has been blurred by dataset‐specific analytical criteria. Taxa designated as ‘core’ were highly sensitive to methodological choices and often reflected explanatory value rather than conserved biological membership. Moreover, many studies that identify taxonomic ‘core’ members interpret their significance in functional terms, suggesting that functional conservation may be the biological interest. Taxonomic conservation may not be the most biologically meaningful target in highly heterogeneous soil and rhizosphere systems, where functional conservation may persist despite taxonomic turnover. Accordingly, ‘core microbiome’ should be reserved for microbial components explicitly demonstrated to be conserved across defined spatial, temporal, and environmental dimensions and linked to conserved ecological functions, while taxa selected for explanatory value are better described as ‘explanatory subsets of taxa’. Greater terminological precision will improve cross‐study comparability and strengthen ecological inference in plant–soil microbiome research.

## Graphical abstract

Soil microbiome studies often define ‘core microbiomes’ using different operational criteria, including occurrence thresholds, abundance thresholds, or other selection methods. Using 1813 soil samples collected across four U.S. Department of Energy Bioenergy Research Centers, we show that these different operational choices identify different subsets of taxa, highlighting that operationally defined subsets should not automatically be interpreted as conserved core microbiomes. Instead, such subsets are better viewed as explanatory subsets of taxa selected for specific analytical purposes, providing a clearer conceptual framework for interpreting soil microbiome datasets.

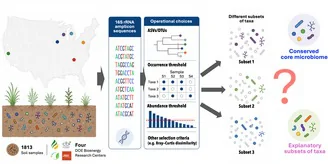

## Introduction

During a 2024 workshop bringing together researchers from the four US Department of Energy Bioenergy Research Centers (BRCs), we attempted to identify a shared core microbiome across rhizosphere soils associated with miscanthus, poplar, sorghum, and switchgrass. Our goal was to identify this core set of taxa to facilitate the construction of synthetic microbial communities that could promote plant growth and stress tolerance. While our analyses did not reveal a substantial taxonomic core shared across sites or systems, the collaborative process surfaced a deeper issue: the field often uses the term ‘core microbiome’ without consistent conceptual and operational definitions or clear specification of the spatial, temporal, and environmental context. Here, we reflect on lessons from our cross‐center efforts and advocate for more deliberate use of the term ‘core’ in microbiome research. Specifically, we argue that core microbiomes should be anchored to clearly defined spatial, temporal, and environmental dimensions and tied to functional relevance. Defining these dimensions clarifies the context in which conservation is being evaluated. Together, these considerations help distinguish biologically conserved components from taxa selected through study‐specific analytical criteria.

## Revisiting the original concept of the core microbiome

The concept of a core microbiome gained prominence through host‐associated microbiome research, particularly during the early era of the Human Microbiome Project, where it was framed as the set of microbial members or functions consistently shared across individuals within a defined habitat (Turnbaugh *et al*., 2007; Lloyd‐Price *et al*., 2016). Even in human‐associated systems, operational thresholds were unavoidable. However, the conceptual expectation of conservation was explicit and tied to a clearly bounded habitat. Importantly, early work emphasized that such conservation was rarely captured at fine taxonomic resolution and was instead more robustly observed at the level of functional genes or metabolic pathways, reflecting conserved biological functions rather than shared species identities (Turnbaugh *et al*., 2009; Lloyd‐Price *et al*., 2016; Kokou *et al*., 2019; Berg *et al*., 2020).

From this original formulation, four conceptual characteristics of a core microbiome can be identified: high prevalence across individual samples, niche‐specific adaptation, temporal stability, and functional convergence (Table 1; Turnbaugh *et al*., 2009; Chakraborty *et al*., 2025). These characteristics serve as a conceptual anchor for clarifying what the term ‘core’ implies, rather than as a checklist. A core microbiome should not simply denote taxa that are frequent in a dataset; instead, it should refer to microbial components that are consistently observed within a defined system, repeatedly selected by a particular habitat or niche, persistent across relevant temporal scales, and linked to conserved ecological functions.

**Table 1:** Key characteristics of a core microbiome.

Characteristic	Description	References
High prevalence across individual samples	Core members should be consistently observed across most individuals within the defined system.	Turnbaugh *et al*. (2007); Turnbaugh *et al*. (2009); Kokou *et al*. (2019); Risely (2020)
Niche‐specific adaptation	Core members should be associated with, adapted to, or repeatedly selected within a particular habitat rather than occurring by chance.	Turnbaugh *et al*. (2007); Lloyd‐Price *et al*. (2016); Kokou *et al*. (2019)
Temporal stability	Core members should persist through time rather than represent transient colonizers.	Faith *et al*. (2013); Kokou *et al*. (2019); Risely (2020)
Functional convergence	Core microbiomes should be associated with conserved ecosystem functions.	Turnbaugh *et al*. (2009); Lloyd‐Price *et al*. (2016); Risely (2020)

## Uses of the ‘core microbiome’ concept in soil and plant‐associated studies

As the concept has been adopted in environmental and plant‐associated microbiome studies, its meaning has gradually shifted. In practice, the term ‘core microbiome’ is now frequently used to describe taxa that are detected in many samples, relatively abundant, or statistically associated with host plants, treatments, or environments of interest, often defined using study‐specific occurrence or abundance thresholds (Shade & Stopnisek, 2019; Risely, 2020; Custer *et al*., 2023). While such operational definitions are analytically meaningful and can highlight informative subsets of the community, they differ from the original intent of the core microbiome concept. Rather than identifying microbial components consistent with the original concept, many studies use ‘core microbiome’ for taxa selected by study‐specific analytical criteria.

Such broadened usage is also apparent in recent soil‐ and plant‐associated microbiome studies (Supporting Information Table S1). In some studies, a core microbiome conceptually refers to taxa that persist across many individual samples, locations, seasons, or host genotypes (Stopnisek & Shade, 2021; Qiu *et al*., 2022; Dasgupta *et al*., 2024). In other studies, the term emphasizes broader geographic or system‐level sharedness, with core taxa defined as those detected at least once in each site or region (Kumar *et al*., 2023; Klasek *et al*., 2024). The term is also used for taxa inferred to be important for host performance, nutrient cycling, ecosystem multifunctionality, or plant health (Liu *et al*., 2023; Eberly *et al*., 2024; Sun *et al*., 2024). In another set of studies, ‘core’ refers to taxa associated with particular treatments or environmental conditions, or taxa selected through network analyses and predictive models because they explain variation in a dataset (Zhong *et al*., 2023; Chen *et al*., 2024; Zhou *et al*., 2024).

Operational thresholds also vary even among studies that share a similar conceptual basis. For example, sample‐level persistence‐based studies have defined core membership as detection in every sample within a dataset (Dasgupta *et al*., 2024), detection across all samples spanning multiple regions (Stopnisek & Shade, 2021), detection in ≥ 80% of sites (Sveen *et al*., 2024), or detection in ≥ 25% of samples across multiple countries (Simonin *et al*., 2020). Other studies combine occurrence and abundance criteria, such as requiring members to belong to the top 10% most abundant taxa while also occurring in > 80% of samples (Guo *et al*., 2024), or to occur in ≥ 50% of samples while exceeding a minimum relative‐abundance threshold (Abera *et al*., 2022). These operational differences are compounded by differences in sequence resolution, with some studies defining taxa as amplicon sequence variants (ASVs) and others using operational taxonomic units (OTUs) clustered at thresholds such as 97% sequence similarity (Table S1).

This variation raises a question for soil and rhizosphere studies: do current uses of ‘core microbiome’ reflect the key characteristics associated with the original concept, including recurrence, niche specificity, temporal stability, and functional convergence? This question is especially important for soil and rhizosphere systems, where microbial communities are not organized around discrete, genetically bounded ecological units, but instead reflect spatially heterogeneous assemblages shaped by plant traits, microenvironmental variation, dispersal, and stochastic processes (Vos *et al*., 2013; Zhou & Ning, 2017).

## Searching for a shared taxonomic core across bioenergy systems

Despite the issues outlined above, the search for shared or recurrent taxa in soil or rhizosphere communities remains useful as a practical starting point for reducing community complexity, supporting cross‐study synthesis, and identifying microbial indicators linked to ecosystem processes or plant performance. Our cross‐BRC effort began from the same motivation: could a shared rhizosphere core microbiome be identified across bioenergy systems that differ in geography, crop type, and management history?

Here, we use the term ‘taxa’ in a broad operational sense to refer to sequence‐defined microbial units (e.g. ASVs or OTUs). These units are useful for community analysis, but their frequency within a dataset does not automatically imply biological conservation or functional importance. This distinction is critical for interpreting taxonomically defined ‘cores’ in highly diverse and heterogeneous soil systems.

Soil and rhizosphere microbial communities exhibit extraordinary taxonomic diversity, strong spatial heterogeneity, and large reservoirs of low‐abundance members (Torsvik & Øvreås, 2002; Delgado‐Baquerizo *et al*., 2018). As taxonomic resolution increases, finer‐scale variation among samples becomes more apparent rather than converging (Tikhonov *et al*., 2015). Under these conditions, instability in taxonomically defined ‘core’ membership is not unexpected and may reflect fundamental properties of rhizosphere microbial community structure.

To evaluate whether a shared core microbiome could be identified across bioenergy systems, we merged 16S rRNA gene amplicon datasets collected from rhizosphere soils spanning multiple U.S. sites, seasons, and management contexts (Fig. 1). At the phylum level, compositional patterns were broadly similar across crops and locations, with dominant groups consistently represented across systems. However, this similarity diminished under stricter taxonomic resolution. At the ASV level, overlap was extremely limited. No ASV was universally present across all samples, and only five ASVs, representing less than 0.01% of all observed ASVs, occurred in more than 75% of samples. Relaxing clustering thresholds increased apparent similarity, consistent with evidence that sequence identity cutoffs are inherently data‐dependent rather than fixed (Edgar, 2018). This result raised a further question: was the weak taxonomic overlap a biological pattern or a consequence of the operational threshold used to define ‘core’?

**Fig. 1:**
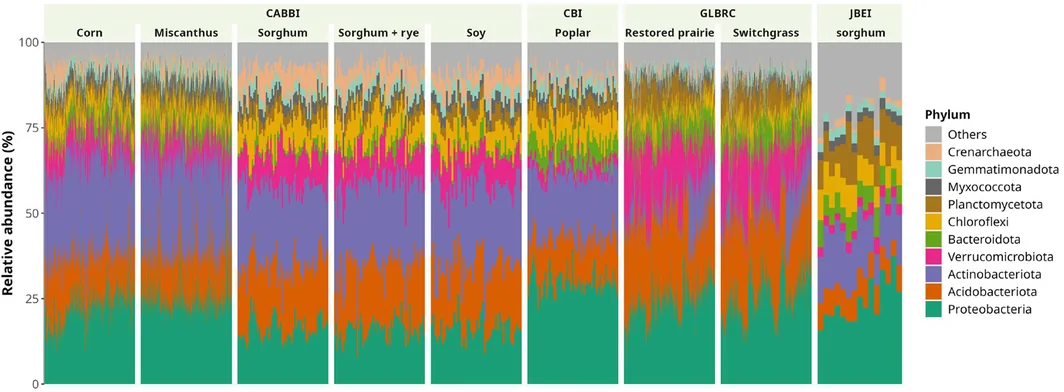
Broad phylum‐level composition of soil bacterial communities across bioenergy cropping systems from four US Department of Energy Bioenergy Research Centers (BRCs). Stacked bar plots show relative abundances of dominant bacterial phyla across 1813 soil samples collected from Center for Advanced Bioenergy and Bioproducts Innovation (CABBI), Center for Bioenergy Innovation (CBI), Great Lakes Bioenergy Research Center (GLBRC), and Joint BioEnergy Institute (JBEI) field sites, representing multiple crops, locations, and management contexts. Phyla contributing lower relative abundance were grouped as Others. At coarse taxonomic resolution, similar phylum‐level groups are detected across systems, although their relative abundances vary among sites and crops.

## Core identification is highly sensitive to methodological choices

The weak ASV‐level overlap could be read in two ways: either a conserved taxonomic core is limited or absent in these systems, or ‘core’ membership is partly shaped by how it is operationally defined. This dependence on operational definition becomes explicit when occurrence thresholds and clustering criteria are systematically varied. As shown in Fig. 2, the number of taxa classified as ‘core’ declines sharply as occurrence thresholds increase and shifts substantially across clustering thresholds (85–100% sequence similarity). The proportion of total sequences represented by these taxa also declines, but to a lesser extent under relaxed definitions. While operational definitions are often acknowledged as necessary, our analyses demonstrate how strongly inferred ‘core’ membership shifts under even small analytical changes (Tikhonov *et al*., 2015; Mysara *et al*., 2017; Abera *et al*., 2022).

**Fig. 2:**
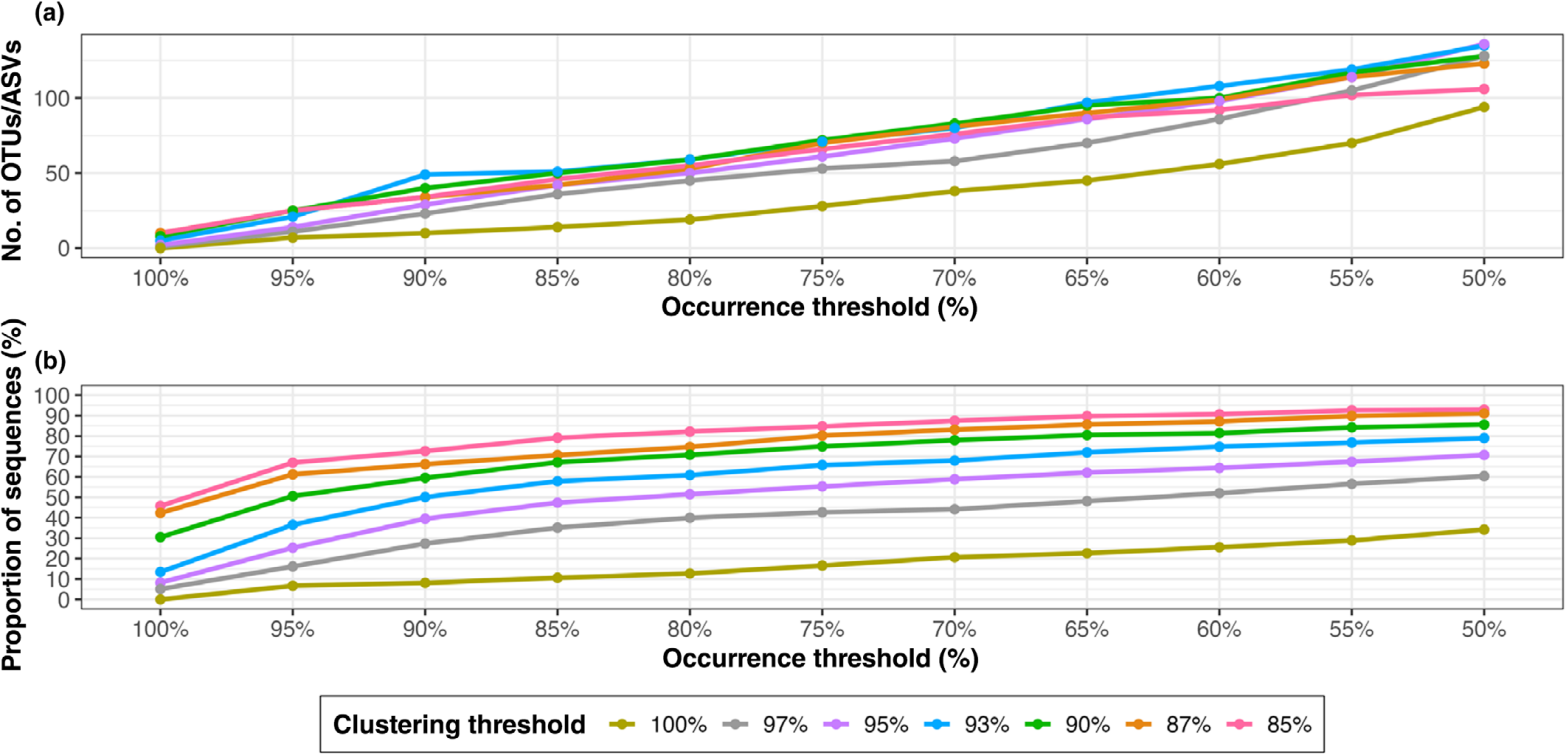
Sensitivity of taxonomic ‘core’ definition to clustering identity and occurrence thresholds. Soil bacterial communities were analyzed from 213 samples collected across 15 small plots at the Sustainable Advanced Bioeconomy Research (SABR) farm located in Ames, Iowa, during the 2023 growing season. Amplicon sequence variants (ASVs; 16 879 unique sequence variants) were obtained using QIIME 2 (Bolyen *et al*., 2019) and subsequently clustered at 85–97% sequence identity using VSEARCH to generate OTU‐level datasets (Rognes *et al*., 2016). (a) Number of taxa classified as ‘core’ across combinations of clustering thresholds and occurrence cutoffs (50–100%). (b) Proportion of total sequences represented by taxa classified as ‘core’ under the same parameter combinations. Colored lines represent the different clustering identity thresholds indicated in the figure key. As occurrence thresholds and clustering thresholds increase, the number of taxa meeting ‘core’ criteria declines sharply. However, despite substantial variation in taxonomic membership, taxa classified as ‘core’ consistently represent a large fraction of total sequence abundance under relaxed definitions. These results illustrate how modest analytical changes substantially alter selected core size and composition, while abundance‐weighted representations can obscure the definition‐dependence of taxonomic core membership.

## A conceptual tension: recurrence vs explanation

At the same time, many studies implicitly evaluate ‘core’ taxa based on their ability to explain variation among samples or treatments. This expectation can introduce a conceptual tension as it relies on an assumption that taxa observed frequently or consistently will also explain variation. Notably, a taxon may be persistent or widespread yet contribute little to between‐sample dissimilarity when its abundance remains relatively stable across samples (Neu *et al*., 2021). Conversely, taxa that strongly differentiate treatments may be neither widespread nor conserved. The search for a shared core brings together two analytical objectives that are often treated as interchangeable: identifying recurrent membership and optimizing explanatory reconstruction of community differentiation. Although these objectives are not necessarily mutually exclusive, they are conceptually distinct (Neu *et al*., 2021).

## Dissimilarity‐based selection does not resolve the conceptual ambiguity of ‘core’

To evaluate alternative frameworks for identifying a shared core microbiome, we compared occurrence‐based definitions with Bray–Curtis dissimilarity‐based (BC‐based) selection. The former prioritizes frequently observed taxa, and the latter prioritizes taxa that contribute to between‐sample variation. Taxa selected using BC‐based criteria (i.e. using a ≥ 2% successive‐increase criterion in cumulative Bray–Curtis dissimilarity) occupy overlapping regions of abundance‐occupancy space with those selected under lower occurrence thresholds (Fig. 3). As the occurrence cutoff is lowered (e.g. from 60 to 40%), the proportion of BC‐selected ASVs captured by occurrence‐based definitions increases progressively.

**Fig. 3:**
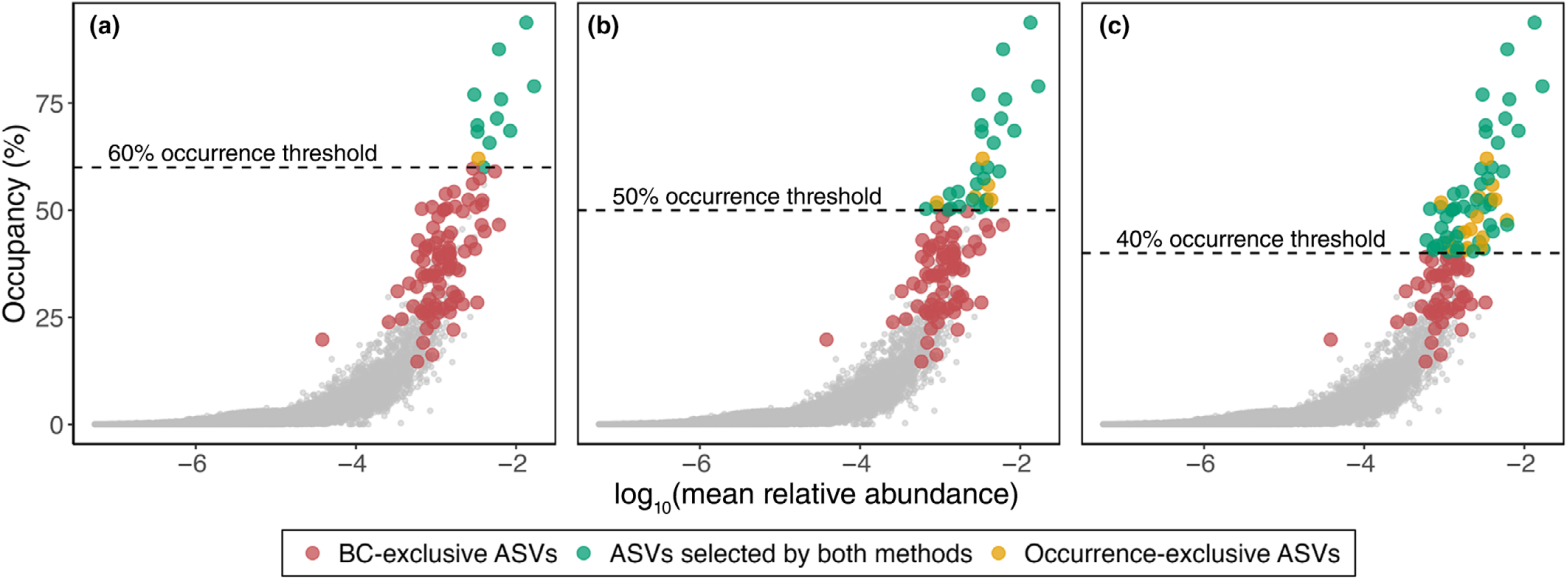
Comparison of occurrence‐based and Bray–Curtis‐based (BC‐based) selection within abundance‐occupancy space. Analyses were conducted on a merged 16S rRNA gene amplicon dataset comprising 1813 soil samples collected from bioenergy cropping systems across the four US Department of Energy Bioenergy Research Centers (BRCs). After quality control, removal of singletons and nonbacterial sequences, and filtering for minimum read depth (≥ 100 reads per sample) and amplicon sequence variant (ASV) abundance (≥ 20 reads), 59 961 ASVs remained for downstream analyses. ASVs selected using BC‐based criteria (i.e. a ≥ 2% successive‐increase criterion in cumulative Bray–Curtis dissimilarity following rarefaction to 5000 reads per sample) were compared to taxa selected under occurrence thresholds of (a) 60%, (b) 50%, and (c) 40%. Each point represents an ASV positioned in abundance‐occupancy space (*x*‐axis: log_10_ mean relative abundance; *y*‐axis: occupancy across samples). Red points indicate ASVs selected exclusively by the BC‐based approach, yellow points indicate ASVs selected exclusively by the occurrence threshold, green points indicate ASVs selected by both methods, and gray points indicate ASVs selected by neither approach. [Correction added on 29 August 2026, after first online publication: the descriptions of the yellow and green points in the preceding sentence have been updated.] Dashed horizontal lines indicate the corresponding occurrence thresholds for each panel. BC‐selected ASVs consistently occupy the upper‐right region of abundance‐occupancy space and overlap increasingly with occurrence‐selected ASVs as the occurrence threshold is lowered. These results demonstrate that BC‐based and occurrence‐based approaches represent alternative operational strategies for selecting taxa within the same underlying distribution.

When BC‐based selection is applied, it does not necessarily recover all highly abundant taxa but rather prioritizes those taxa whose variation in abundance contributes most to community dissimilarity (Shade & Stopnisek, 2019). Nevertheless, the taxa selected through this approach fall along the same abundance‐occupancy continuum as those identified under threshold‐based definitions. Thus, BC‐based selection can be understood as an alternative way of prioritizing taxa within the same underlying abundance‐occupancy distribution, as Bray–Curtis dissimilarity is derived directly from the community abundance matrix and can be partitioned into taxa‐level contributions (Clarke, 1993; Legendre & De Cáceres, 2013; Shade & Stopnisek, 2019). Accordingly, shifting from occurrence thresholds to dissimilarity‐based selection does not resolve the conceptual ambiguity of ‘core’.

## Subsets of taxa can enhance explanatory power without being ‘core’

The distinction between a core microbiome and an analytically selected taxa subset does not make subset selection uninformative. Subset selection can improve inference when aligned with a specific ecological question. For example, using a *Miscanthus* × *giganteus* field experiment, occurrence‐based taxa subsets were compared with the full community and randomly selected taxa subsets for their ability to detect fertilization and temporal effects. As shown in Box 1, subsets defined using moderate occurrence thresholds (e.g. 40%) increased PERMANOVA pseudo‐*F* statistics relative to the full community and to random subsets (Fig. 4; Table 2). For temporal effects, explanatory power nearly doubled compared to the full community.

**Fig. 4:**
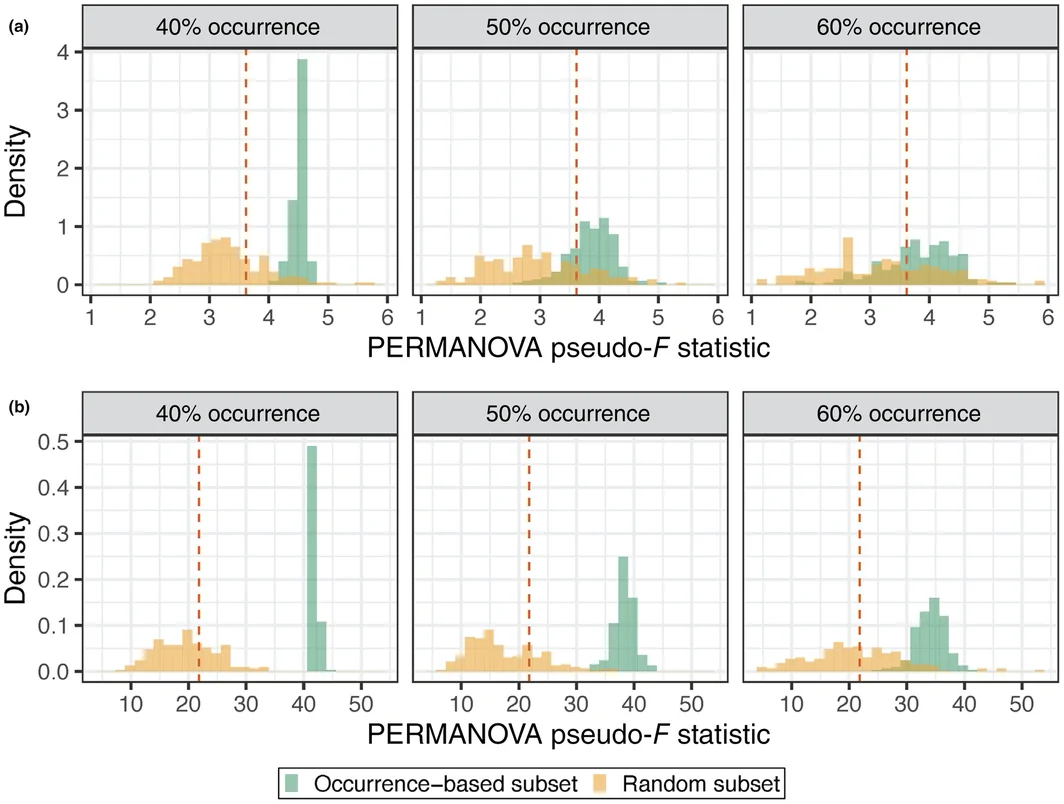
Context‐defined occurrence‐based taxa subsets can increase sensitivity for detecting fertilization and temporal effects in M × g soil microbial communities. Density distributions show PERMANOVA pseudo‐*F* statistics from 200 bootstrap iterations comparing occurrence‐based taxa subsets selected at 40%, 50%, and 60% occurrence thresholds with randomly selected taxa subsets of equivalent size. Community differences were calculated using Bray–Curtis dissimilarities, with significance assessed using 999 permutations. (a) Detection of nitrogen fertilization effects. (b) Detection of stand‐age effects. Green distributions represent occurrence‐based subsets, orange distributions represent randomly selected subsets, and vertical dashed lines indicate the pseudo‐*F* statistic obtained using the full community dataset.

**Table 2:** Gains in statistical resolution from context‐defined subsets of M × g soil microbial communities compared with the full dataset and randomly selected taxa, as measured by the PERMANOVA pseudo‐*F* statistics for fertilization and stand age effects (Δ vs Full, percentage change relative to the full dataset; Δ vs Random, percentage change relative to the mean of randomly selected taxa subsets of equivalent size).

Subset type	Threshold	Pseudo‐*F* stat	Δ vs Full	Δ vs Random
Fertilization	Full dataset	3.62	–	–
	40%	4.52	25.1%	40.1%
	50%	3.87	6.9%	39.0%
	60%	3.80	4.5%	30.2%
Temporal	Full dataset	21.8	–	–
	40%	41.8	91.9%	130%
	50%	38.3	75.6%	146%
	60%	34.1	56.2%	91.7%

Box 1 **Case study: context‐defined subsets in *Miscanthus* × *giganteus* soil**

Since our cross‐system analyses did not identify a robust universal taxonomic core, we next evaluated whether defining subsets of taxa within a single, well‐characterized experimental system could provide analytical advantages. Specifically, we examined a long‐term *Miscanthus* × *giganteus* (M × g) field experiment designed to test the effect of stand age and nitrogen fertilization (Tejera *et al*., 2019; Ma *et al*., 2021). M × g is a perennial bioenergy grass capable of producing high biomass yields for over a decade without replanting, making it well suited for studying plant–microbe interactions across temporal and management settings. Soil samples were collected across multiple growing seasons and fertilization treatments, enabling us to evaluate whether the subsets of frequently observed taxa can improve the ability to detect treatment effects relative to the whole community.

Rather than attempting to define a universal core, we constructed context‐defined subsets representing taxa consistently detected across specific experimental conditions. Two types of subsets were defined: (1) a fertilization‐stable subset, consisting of taxa consistently present across nitrogen treatments and (2) a temporal stable subset, consisting of taxa consistently present across stand ages.

For each subset type, we applied three occurrence thresholds (presence in 40%, 50%, or 60% of samples). To assess whether these subsets provided analytical advantages, we compared their performance to (1) the complete community dataset and (2) randomly selected taxa of equivalent size. Random subsets were generated using a bootstrapping approach (200 iterations). Community differences were evaluated using permutational multivariate analysis of variance (PERMANOVA) with the *adonis2()* function from the vegan package, v.2.6‐10 (Oksanen *et al*., 2025), based on the Bray–Curtis dissimilarity distances between samples with *P*‐values for the test statistic (pseudo‐*F*) with 999 permutations.

Using the full dataset established baseline effect sizes: both nitrogen fertilization (*R* ^2^ = 0.014, *P* = 0.001) and stand age (*R* ^2^ = 0.085, *P* = 0.001) explained a small but statistically significant portion of beta diversity. Subsets defined at a lower occurrence threshold (particularly 40%) increased the PERMANOVA pseudo‐*F* statistic relative to both the full dataset and random subsets (Table 2). For temporal effects, explanatory power nearly doubled compared to the whole community. However, higher occurrence thresholds (50% and 60%) reduced this advantage, suggesting that explanatory performance itself is threshold‐sensitive (Fig. 4). These results show that context‐defined subsets can enhance statistical resolution for specific research questions. Notably, these improvements reflect analytical optimization within a defined system but do not provide evidence of conserved biological membership across spatial or environmental contexts.

This case study demonstrates a major distinction emphasized throughout this manuscript: subsets of taxa can improve explanatory performance within a specific dataset, yet such subsets may not represent a biologically conserved ‘core’ microbiome. Analytical usefulness and cross‐context persistence represent fundamentally different concepts.

More restrictive thresholds reduced these gains, demonstrating that explanatory performance is itself threshold‐sensitive. Thus, subset selection can function as a dimensionality‐reduction strategy that improves treatment resolution within a defined experimental context. Such subsets are selected for explanatory value rather than for evidence of conserved membership across spatial, temporal, or environmental dimensions.

## Taxonomic conservation may not be the biologically meaningful target

Across the studies summarized in Table S1, taxonomic ‘core’ assignments were frequently linked to community stability, nutrient cycling, disease suppression, plant health, ecosystem multifunctionality, carbon dynamics, or other ecological outcomes (Qiu *et al*., 2022; Liu *et al*., 2023; Guo *et al*., 2024; Sun *et al*., 2024; Zhou *et al*., 2024). For example, ‘core’ taxa have been linked to cotton disease resistance and pathobiome structure (Qiu *et al*., 2022), soil carbon, nitrogen, and phosphorus cycling and wheat productivity (Liu *et al*., 2023), residue decomposition and soil multifunctionality in maize systems (Guo *et al*., 2024), soil ecosystem multifunctionality under coppice planting (Sun *et al*., 2024), and soil carbon dynamics under long‐term fertilization (Zhou *et al*., 2024). Yet these interpretations were rarely supported by direct measurements of conserved microbial functions. Instead, functional importance was typically inferred from functional prediction tools such as PICRUSt2, FungalTraits, FAPROTAX, Tax4Fun2, FUNGuild, or PBB annotations (Liu *et al*., 2023; Chen *et al*., 2024; Dasgupta *et al*., 2024; Eberly *et al*., 2024; Guo *et al*., 2024; Nutaratat *et al*., 2024), network or keystone‐taxa analyses (Qiu *et al*., 2022; Dasgupta *et al*., 2024; Zhou *et al*., 2024), statistical associations or structural equation modeling (Liu *et al*., 2023; Chen *et al*., 2024; Guo *et al*., 2024; Sun *et al*., 2024; Zhou *et al*., 2025), or taxonomic identity and literature‐supported traits (Kumar *et al*., 2023; Zhong *et al*., 2023). This pattern was evident across conceptual definitions of ‘core’, regardless of whether taxa were selected based on persistence, geographic sharedness, host association, treatment response, or predictive importance.

This recurring pattern suggests that the primary scientific interest is often not taxonomic conservation itself, but whether ecological functions remain conserved across samples, environments, or hosts. In this same literature, some studies acknowledge that amplicon‐based taxonomic data alone cannot establish functional roles and recommend metagenomic, cultivation‐based, or experimental approaches to determine whether proposed core taxa contribute to ecosystem processes or host performance (Simonin *et al*., 2020; Stopnisek & Shade, 2021; Eberly *et al*., 2024). In this sense, many discussions of a ‘core microbiome’ appear to be motivated by an interest in functional conservation, even when the analytical object being labeled as ‘core’ remains taxonomic.

Viewed from this perspective, the limited evidence for a conserved taxonomic core across heterogeneous soil and rhizosphere systems may indicate that taxonomic conservation is not the most biologically meaningful target in these systems. Functional convergence may persist despite taxonomic turnover, a pattern previously observed in human‐associated microbiomes and other microbial ecosystems (Turnbaugh *et al*., 2009; Lloyd‐Price *et al*., 2016; Kokou *et al*., 2019). The central question is therefore not how taxonomic thresholds should be further refined, but whether ecological functions are conserved across spatial, temporal, and environmental contexts.

## Toward greater precision in the use of ‘core microbiome’

Our analyses highlight a conceptual distinction that is frequently conflated in the literature (Shade & Handelsman, 2012). Taxa identified as ‘core’ under threshold‐based or BC‐based frameworks may reflect analytical prioritization within a broad abundance‐occupancy space. These taxa may be frequent, abundant, or influential in reconstructing patterns of community differentiation. These objectives can be analytically legitimate and often useful, yet they differ from the original biological meaning of a core microbiome. Thus, not every subset labeled as ‘core’ represents a core microbiome.

Two distinct concepts are frequently conflated under the single term ‘core microbiome’: (1) microbial components linked to ecological functions and conserved across clearly specified spatial, temporal, and environmental scales and (2) taxa selected within a dataset because they optimize explanatory power or reconstruction of community differentiation under specific analytical criteria. The latter represents an optimization strategy within abundance‐occupancy space and is inherently dependent on methodological positioning. We refer to these analytically selected groups as ‘explanatory subsets of taxa’. If the objective is to identify a biologically conserved core microbiome, then threshold‐based or dissimilarity‐based selection alone may be insufficient.

In conclusion, we propose greater terminological precision. The term ‘core microbiome’ should be used only when microbial components show conservation across clearly defined spatial, temporal, and environmental dimensions with functional relevance explicitly supported rather than assumed. Where the analytical objective is to enhance explanatory power or reconstruct community differentiation within a dataset, the resulting taxa are more accurately described as explanatory subsets of taxa. Adopting this distinction preserves the analytical value of subset‐based approaches while restoring conceptual clarity. By separating conserved members, conserved functions, and explanatory subsets of taxa, microbiome research can enhance cross‐study comparability, strengthen ecological interpretation, and reduce ambiguity in the use of the term ‘core’.

## Competing interests

None declared.

## Author contributions

JL contributed to conceptualization, methodology, software, validation, formal analysis, investigation, data curation, writing – original draft, writing – review and editing. BAR contributed to conceptualization, methodology, software, validation, formal analysis, investigation, data curation. PL contributed to conceptualization, methodology, software, validation, formal analysis, investigation, data curation. GMNB contributed to conceptualization, methodology, software, validation, writing – review and editing. YNC contributed to conceptualization, methodology, software, validation, data curation. BK contributed to conceptualization, methodology, software, validation, writing – review and editing. MAC, AH, SL and HVS contributed to conceptualization, methodology, validation, resources, writing – original draft, writing – review and editing, supervision. MAC, AH, SL and HVS contributed equally to this work. JMA and TAR contributed to conceptualization, writing – review and editing. AAC, JC, NG, VI, SM, IP, RP, LR, NSS, AS and PFS contributed to conceptualization.

## Disclaimer

The New Phytologist Foundation remains neutral with regard to jurisdictional claims in maps and in any institutional affiliations.

## Supporting information


**Table S1** Representative examples of conceptual and operational definitions used for ‘core microbiome’ assignments in recent plant‐ and soil‐associated microbiome studies.
Please note: Wiley is not responsible for the content or functionality of any Supporting Information supplied by the authors. Any queries (other than missing material) should be directed to the *New Phytologist* Central Office.

## Data Availability

All datasets and code used for this article are available at https://github.com/germs‐lab/rethinking‐soil‐core‐microbiome‐analysis. The raw data used for this analysis are deposited at the National Microbiome Data Collaborative within the following study identifiers: nmdc:sty‐11‐n7mtj961, nmdc:sty‐11‐wbc14h22, nmdc:sty‐11‐e4yb9z58, nmdc:sty‐11‐kfvmk798, and nmdc:sty‐11‐vh2hty57.
